# Systemic lupus erythematosus associated with paroxysmal nocturnal hemoglobinuria: a case report and literature review highlighting the clinical significance of small PNH clones

**DOI:** 10.3389/fimmu.2026.1859655

**Published:** 2026-06-26

**Authors:** Xiaoyan Huang, Jing Huang, Ning Liu, Lijun Zhang

**Affiliations:** 1 Department of Rheumatology, The University of Hong Kong – Shenzhen Hospital, Shenzhen, China; 2 Shenzhen Clinical Research Center for Rare Diseases, Shenzhen, China; 3 Department of Hematology, The University of Hong Kong – Shenzhen Hospital, Shenzhen, China

**Keywords:** case report, cytopenia, hemolysis, paroxysmal nocturnal hemoglobinuria, systemic lupus erythematosus, complement inhibitors

## Abstract

Systemic lupus erythematosus (SLE) and paroxysmal nocturnal hemoglobinuria (PNH) represent distinct disorders linked by complement pathway dysregulation, yet their co-occurrence remains poorly characterized. We present a 42-year-old woman with newly diagnosed SLE exhibiting persistent cytopenia and Coombs-negative hemolysis, in whom high-sensitivity flow cytometry identified GPI-deficient clones consistent with PNH. Following treatment for SLE, she showed clinical improvement with stable small granulocyte PNH clone during more than 2 years of follow-up. Systematic literature review of seven additional cases reveals that SLE-associated PNH manifests heterogeneously, with thrombotic events occurring in 37.5% (3/8) of patients and detectable hemolysis in 87.5% (7/8). Notably, our analysis challenges the conventional view that small PNH clones (<10% granulocytes) are clinically insignificant - two such cases demonstrated biochemical evidence of hemolysis. These findings suggest that even small PNH clones may contribute to clinical hemolysis in SLE patients through autoimmune complement activation. This observation has immediate clinical relevance, as it justifies PNH screening in SLE patients with unexplained cytopenia or Coombs-negative hemolysis, and the management should be guided by clinical phenotype rather than clone size alone.

## Introduction

Systemic lupus erythematosus (SLE) is a prototypic autoimmune disease marked by immune dysregulation and complement activation. Hematologic abnormalities are frequently seen, including cytopenia, hemolysis and thrombosis. As these manifestations are well recognized in SLE and incorporated into classification criteria, they are often interpreted within the spectrum of disease activity. However, alternative or concomitant hematologic disorders may occasionally coexist and remain underrecognized.

Paroxysmal nocturnal hemoglobinuria (PNH) is a rare acquired clonal hematopoietic stem cell disorder caused by somatic mutations in the *PIGA* gene, resulting in deficiency of glycosylphosphatidylinositol (GPI)-anchored proteins such as CD55 and CD59. The absence of these complement regulatory proteins predisposes erythrocytes to complement-mediated hemolysis and is associated with varying degrees of bone marrow failure and thrombotic risk (1). Clinical manifestations are highly heterogeneous and are generally associated with both clone size and complement activity, ranging from asymptomatic small clones to classical hemolytic disease with significant morbidity. High-sensitivity multiparameter flow cytometry, particularly with fluorescent aerolysin (FLAER), has become the diagnostic standard and allows detection of very small PNH clones (2). Small and very small PNH clones are frequently detected in bone marrow failure spectrum diseases as “survivor populations” under the immune stress, and the presentation is usually associated with better prognosis (3).

Given the shared features of immune dysregulation and complement activation, the coexistence of SLE and PNH is biologically plausible. Immune-mediated bone marrow stress, a well-recognized mechanism in PNH clonal expansion, may also occur in autoimmune conditions and provide a permissive environment for the emergence or persistence of PNH clones. However, despite this mechanistic link, the coexistence of SLE and PNH has been rarely reported, and its clinical characteristics remain poorly defined.

In this study, we report a case of SLE associated with a small PNH clone accompanied by clinically significant hemolysis. We further performed a literature review of previously published reports to analyze the clinical manifestations, PNH clone size, treatment strategies, and outcomes in patients with SLE–PNH overlap. Based on our findings, we propose that small PNH clones, although traditionally considered clinically silent, may contribute to hemolysis in the context of SLE, highlighting the importance of recognizing this underappreciated overlap in clinical practice.

## Case presentation

A 42-year-old woman was admitted to our hospital in April 2023 for evaluation of persistent pancytopenia that had been present for more than five years. Five years before admission, routine laboratory testing at another hospital revealed leukopenia (white blood cell count 3.78×10^9^/L), severe anemia (hemoglobin 53 g/L), and thrombocytopenia (platelet count 46×10^9^/L). Bone marrow examination at that time showed active hematopoiesis with occasional megaloblastoid changes in erythroid precursors. The patient was diagnosed with “nutritional anemia” and treated with oral iron and folic acid, resulting in partial improvement in blood counts. She did not undergo regular follow-up thereafter.

Approximately two years before admission, she gradually developed alopecia, photosensitivity, and bilateral shoulder arthralgia without joint swelling or morning stiffness. Three months before admission, she experienced left knee pain after minor trauma. Laboratory testing at our hospital on April 3, 2023 demonstrated persistent pancytopenia with a white blood cell count of 3.02×10^9^/L, hemoglobin 83 g/L, and platelet count 66×10^9^/L.

Her past medical, personal, and family histories were unremarkable.

On admission, physical examination revealed slightly thin hair, but no apparent skin rash. No joint swelling or tenderness was observed. Repeat testing confirmed pancytopenia (white blood cell count 2.11×10^9^/L, hemoglobin 69 g/L, platelet count 57×10^9^/L). The mean corpuscular volume was elevated (MCV 102.5 fL), and the reticulocyte percentage was increased (4.77%). Lactate dehydrogenase was mildly elevated (494 U/L), and haptoglobin was markedly decreased (<25 mg/dL), supporting the presence of hemolysis. Urinalysis showed no bilirubin. Serum ferritin, folate, and vitamin B12 levels were within normal ranges. Liver and renal function tests were normal. Immunologic testing revealed a high-titer antinuclear antibody (ANA 1:1000) with a speckled pattern, anti-Sm antibody positivity, and U1-RNP/Sm positivity. The direct Coombs test was negative, and antiphospholipid antibodies were not detected.

According to 2019 ACR/EULAR classification criteria for SLE, the patient fulfilled the ANA entry criterion and reached a total score of 12 points, including anti-Sm antibody positivity (6 points), thrombocytopenia (4 points), and non-scarring alopecia (2 points). Leukopenia was not additionally counted because only the highest-weighted item within the hematologic domain is scored. This clarification supports the diagnosis of SLE.

Given the presence of Coombs-negative hemolysis, high-sensitivity flow cytometry for PNH was performed. The results demonstrated PNH clones involving multiple lineages, including erythrocytes (type II 3.6%, type III 5.6%), granulocytes (5.9%), and monocytes (27.9%). Abdominal ultrasonography revealed no hepatosplenomegaly.

Based on the clinical manifestations and immunological findings, the patient was diagnosed with SLE accompanied by a small PNH clone with laboratory evidence of hemolysis. SLE-directed therapy was prioritized because the patient had active autoimmune features and immune-mediated cytopenia, whereas the PNH clone was small, hemolysis was mild, and there was no thrombosis, transfusion dependence, severe symptomatic hemolysis, or organ-threatening complication. Therefore, complement inhibition was not initiated at presentation. She was instead managed with a regimen of prednisone (1mg/kg/day), cyclosporine (75mg, twice daily), and hydroxychloroquine (200mg, twice daily). Hematologic parameters improved during hospitalization and the patient was discharged.

**Figure 1:**
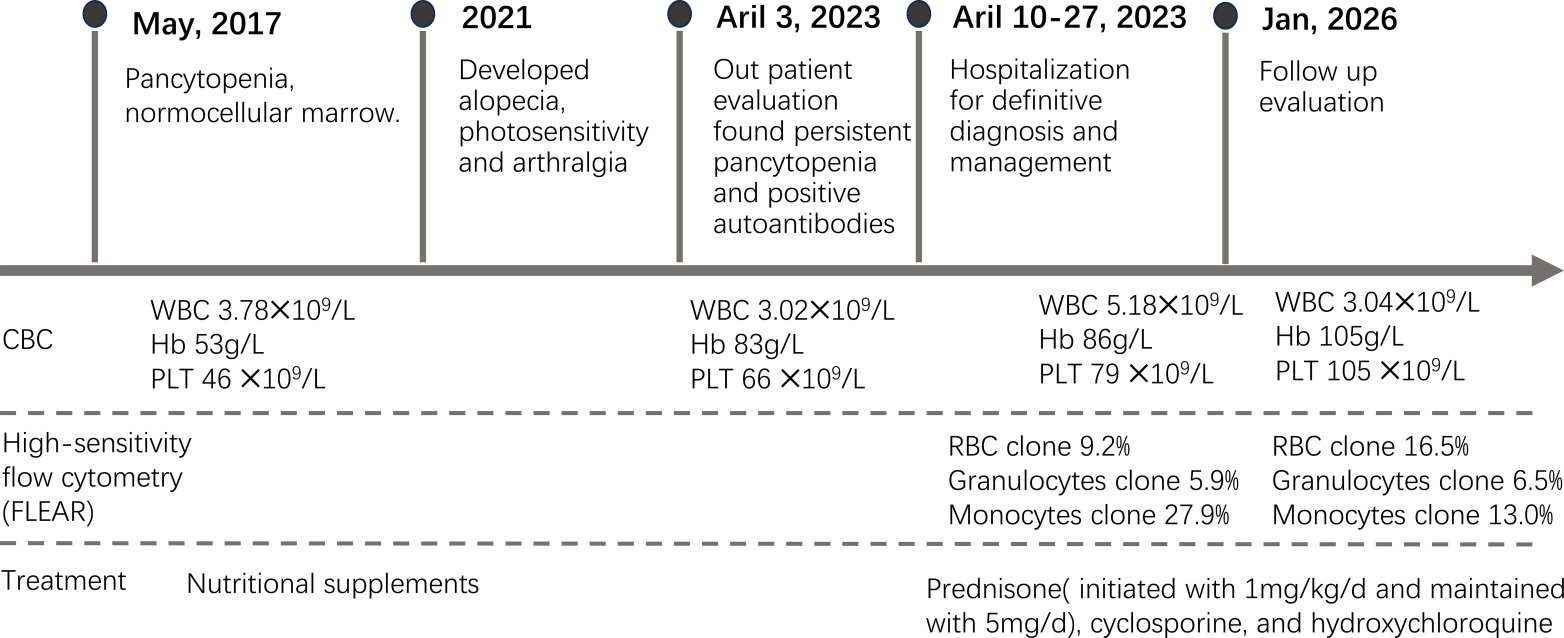
Timeline of clinical course and key laboratory results. CBC, complete blood count; FLAER, flow cytometry-based fluorescent aerolysin assay; Hb, hemoglobin; PLT, platelet count; WBC, white blood cell count.

During follow-up, the patient maintained with low-dose glucocorticoids, cyclosporine, and hydroxychloroquine, she remained clinically stable with persistent mild pancytopenia and no additional organ involvement. The patients tolerated these medications well, and no adverse effects were observed. Repeat high-sensitivity flow cytometry in Jan 2026 demonstrated persistent PNH clones, with erythrocyte type II clones of 3.91%, type III clones of 12.54%, granulocyte clones of 6.5%, and monocyte clones of 12.99% (**Figure 1**).

### Literature search strategy and study selection

A structured literature search was conducted to identify published reports describing SLE complicated by PNH. The databases PubMed, Embase, and Web of Science were searched from inception to March 2026. The following search terms were used in various combinations: “systemic lupus erythematosus” OR “SLE” AND “paroxysmal nocturnal hemoglobinuria” OR “PNH” AND “case report” OR “case series”. Reference lists of relevant articles were manually screened to identify additional eligible reports.

Studies were eligible for inclusion if they met the following criteria: (1) diagnosis of SLE based on established classification criteria at the time of publication; (2) confirmed diagnosis of PNH by flow cytometry demonstrating GPI-anchor deficiency (e.g., CD55/CD59 deficiency or FLAER-based assay); and (3) case report or case series design. Reports without clear confirmation of PNH by flow cytometry, review articles without original cases, conference abstracts lacking sufficient clinical data, and duplicate publications were excluded.

Data extracted from each eligible case included patient demographics, timing of PNH diagnosis relative to SLE, organ involvement, hematological manifestations, autoantibody profile, PNH clone size, treatment strategy (immunosuppressive and/or complement inhibition), and reported outcomes.

## Results

The literature search identified seven published cases eligible for analysis. Together with the present case, a total of eight patients were included (**Table 1**).

**Table 1:** Case summary of SLE associated with PNH.

Author (Year)	Age/Sex	Timing of PNH diagnosis relative to SLE	SLE organ involvement	Hematological manifestations	Autoantibodies	PNH clone size (method)	Bone marrow findings	Treatment (IS/complement inhibition)	Outcome
Gupta et al. (2009) (15)	60 F	3.5 years after SLE diagnosis	Arthralgia, malar rash, proteinuria, chronic hepatitis	Pancytopenia, portal vein thrombosis	Anti-dsDNA positive; low C3/C4; aPLs negative	Granulocytes CD55 deficiency 78%, CD59 deficiency 70%; Ham and sucrose lysis tests positive	Increased megakaryocytes	Supportive care; no complement inhibitor	Clinical improved and stable
Nakamura et al. (2011) (16)	29 F	17 years after SLE onset	Class IV lupus nephritis, arthritis	Pancytopenia, hemolytic anemia, cerebral venous thrombosis	ANA; anti-dsDNA; hypocomplementemia	Reduced RBC CD55 and CD59 expression (18% and 78.6%) (flow cytometry); Ham and sucrose tests positive	Secondary aplastic anemia (reported as background)	Corticosteroids and immunosuppressive therapy; no complement inhibitor	NR
Kontomanolis et al. (2013) (17)	27 F	5 years before SLE	Psychosis	Mild hemolytic anemia, thrombocytopenia, portal vein and hepatic vein thrombosis	ANA, anti-dsDNA, anti-SSA positive; aPLs negative	88% CD55-/CD59-granulocytes(flow cytometry)	Not applicable	Corticosteroids; no complement inhibitor	Improved; favorable pregnancy outcome
Anderson et al. (2018) (4)	70 F	During SLE course with aplastic anemia	Pancytopenia, hemolytic anemia	Pancytopenia, hemolytic anemia	ANA; anti-dsDNA; lupus anticoagulant; positive direct Coombs’ test	>20% PNH clone (flow cytometry)	Polyclonal B lymphocytosis and plasmacytosis, 12 weeks later hypocellular marrow with poikilocytosis and macrocytosis	Corticosteroids, IVIG, rituximab, ATG, cyclosporine; later eculizumab	Hemolysis improved after eculizumab; remained transfusion-dependent
Serin et al. (2020) (18)	30 F	At the time of SLE diagnosis	Alopecia, pleural and pericardial effusion, non-erosive arthritis	Pancytopenia, hemolytic anemia	ANA; anti-dsDNA positive; Coombs test negative	RBC 0.6%; monocytes 7.79%; granulocytes 11.25% (FLAER)	Normocellular marrow, increase in mature plasma cells.	Prednisone, cyclosporine; later rituximab; no complement inhibitor	Blood counts improved with immunosuppression
Hasan et al. (2023) (5)	19 F	At the time of SLE diagnosis	Fever, hair loss, arthralgia, photosensitivity, oral ulcer	Pancytopenia, hemolytic anemia	ANA; direct Coombs negative, indirect Coombs positive	Reduced RBC CD55 and CD59 expression (20.1% and 4.2%)(flow cytometry)	Normal active marrow	Steroids, hydroxychloroquine, no complement inhibitor	Improved
Nakata et al. (2025) (19)	34 F	~5 years after SLE diagnosis	Nephrotic syndrome/lupus nephritis	Pancytopenia, hemolytic anemia	Anti-dsDNA; low C3/C4, Coombs test negative	Initial: RBC 22.1%, granulocytes 24.6%; later high-sensitivity FLAER: RBC(type II and III) 70.1%, granulocytes 88.1%	Normocellular marrow, erythroid hyperplasia; mild granulocytic hypoplasia, minimal megakaryocytes dysplasia	Prednisone, tacrolimus, mycophenolate, belimumab; crovalimab (C5 inhibitor)	Rapid improvement of anemia and LDH; stable
Present case	42 F	At the time of SLE diagnosis	Alopecia, arthralgia	Pancytopenia, hemolytic anemia	ANA, anti-U1RNP and anti-Sm positive; Coombs test negative	RBC 9.2%; monocytes 27.9%; granulocytes 5.9% (FLAER)	Normocellular marrow	Corticosteroid, cyclosporin; no complement inhibitor	Blood counts improved

All reported patients were female, with ages ranging from 19 to 70 years.

The timing of PNH detection varied. PNH developed after the diagnosis of SLE in three patients, occurred concurrently with SLE diagnosis in four patients, and preceded SLE in one patient. Cytopenia were present in all cases, including pancytopenia reported in six patients. Laboratory evidence of hemolysis was described in seven patients. Thrombotic events occurred in three cases, including portal vein thrombosis, hepatic vein thrombosis, and cerebral venous thrombosis.

Regarding the size of PNH clones, the detection methods were not entirely consistent across all these cases. Overall, two cases exhibited small clones, while the remaining six cases demonstrated medium-to-large clones.

Seven cases reported bone marrow findings: normocellular marrow was reported in four cases, aplastic anemia or hypocellular marrow were reported in two cases, and increased megakaryocytes without marrow failure in one case.

Most patients were treated with immunosuppressive therapy for SLE, and hematologic parameters improved in several cases. Complement inhibition was administered in two cases with large clones and clinically significant hemolysis.

## Discussion

The coexistence of SLE and PNH is exceedingly rare, with current evidence limited to isolated case reports. In the present study, the eight cases encompassed classical hemolytic PNH, thrombosis-dominant presentations and cytopenia, highlighting that PNH manifestations in the context of autoimmune disease are not uniform but instead span the full phenotypic range of the disease. As these hematologic abnormalities can also be the manifestations of SLE, and even are included in the classification criteria for diagnosing SLE, the correct attribution is essential for diagnosis and disease management.

In our case, Coombs-negative hemolysis was the trigger for PNH testing, supporting that hemolysis should primarily be attributed to PNH, while the cytopenia is more likely multifactorial based on the relatively active bone marrow appearance, reflecting both immune-mediated marrow injury and clonal hematopoiesis. However, our literature review suggests that the decision to investigate PNH in patients with SLE is also driven by clinical features suggestive of bone marrow failure, intravascular hemolysis, or atypical antiphospholipid antibody negative- thrombosis, rather than Coombs test results alone. For example, Anderson et al. reported a patient with a positive direct Coombs test in whom PNH was ultimately identified during the evaluation of persistent pancytopenia and evolving bone marrow failure (4). In contrast, Hasan et al. described a patient with SLE who presented with dark-colored urine, a manifestation suggestive of intravascular hemolysis and not typically associated with autoimmune hemolytic anemia in SLE (5). This clinical presentation prompted further evaluation, and flow cytometry subsequently confirmed the presence of a PNH clone. Several other cases presented with atypical, anti-phospholipid antibody negative-thrombosis, also prompt the investigation for PNH. These cases illustrate that the trigger for PNH testing may arise from clinical features that are atypical for lupus-related hematologic manifestations, even when autoimmune hemolysis cannot be completely excluded. All these features, including Coombs-negative hemolysis, bone marrow failure, unexplained intravascular hemolysis, and atypical thrombosis, are also recognized as key diagnostic triggers in contemporary PNH practice (6).

The coexistence of SLE and PNH may be explained by the “immune selection” model. PNH arises from somatic mutations in PIGA within hematopoietic stem cells, but these mutations alone are insufficient for clonal expansion. Instead, expansion occurs in the setting of immune-mediated marrow injury, where mutant clones gain a relative survival advantage (7). This paradigm is well established in aplastic anemia and is supported by recent studies demonstrating that PNH clones preferentially expand under immune-mediated bone marrow stress (8–10). Although direct evidence in SLE is limited, the shared features of immune dysregulation suggest that a similar mechanism may operate in autoimmune diseases.

Clone size is traditionally considered a major determinant of PNH clinical phenotype. Large clones are typically associated with overt hemolysis and increased thrombotic risk, whereas small clones are more often observed in bone marrow failure states and are frequently clinically silent (11). Large cohort analyses have demonstrated a positive correlation between clone size and disease burden, including thrombosis risk and laboratory markers of hemolysis (12). However, the relationship is not absolute. Recent studies have shown that even small clones may carry clinical significance, including thrombotic risk and hematologic abnormalities (3). Our case, together with Serin’s, are particularly noteworthy in this context. Despite a small granulocyte clone (5.9% and 11.25%, respectively), both patients exhibited biochemical evidence of hemolysis. This apparent discrepancy may be explained by complement activation driven by immune complexes in SLE, leading to complement-mediated injury of the GPI-deficient erythrocytes. This may suggest that clone size alone may not fully predict clinical phenotype in autoimmune settings.

From a therapeutic perspective, immunosuppressive therapy (IST) remains the cornerstone for SLE, especially those with hematological manifestations. IST may improve cytopenia by alleviating immune-mediated marrow suppression as shown in our case. However, such therapy does not eradicate the PNH clone, so the clone size may increase or fluctuate over time after IST as the hematopoietic environment has been modified (13). This phenomenon suggests that the clone size should be monitored longitudinally, and dynamically evaluate the risk of hemolysis and thrombosis. Therapeutic strategy should be guided by the clinical phenotype rather than clone size alone. Complement inhibition, particularly targeting C5, has become the standard of care for classical PNH in controlling hemolysis and reducing thrombotic risk. Emerging proximal complement inhibitors may further improve outcomes by addressing both intravascular and extravascular hemolysis (14). These therapies may be considered in patients of SLE complicated by PNH even with small or stable clones.

## Patient’s perspective

The following statement was summarized from the patient’s feedback during follow-up. The patient reported that she had experienced abnormal blood counts for several years without a clear explanation. After being diagnosed with systemic lupus erythematosus and a PNH clone, she was concerned but relieved that a more complete explanation had been identified. After treatment with glucocorticoids, cyclosporine, and hydroxychloroquine, she felt reassured by the improvement in blood counts and understood the need for continued follow-up and monitoring of the PNH clone.

## Conclusion

SLE-associated PNH represents a rare but clinically relevant overlap that may be underrecognized in routine practice. Our case and literature review emphasizes that Coombs-negative hemolysis should prompt evaluation for PNH, even when a small clone is detected. Notably, our findings suggest that small PNH clones, traditionally considered clinically insignificant, may contribute to clinically significant hemolysis in the context of autoimmune-mediated complement activation. Treatment strategies should be guided by clinical phenotype rather than clone size alone, with consideration of complement inhibition in selected cases. Further studies are needed to clarify the prevalence, clonal dynamics, and therapeutic implications of PNH in autoimmune diseases.

## Data Availability

The original contributions presented in the study are included in the article/supplementary material. Further inquiries can be directed to the corresponding author.
